# Enhancing emergency department care for individuals with autism spectrum disorder across the lifespan: a systematic review

**DOI:** 10.3389/frhs.2026.1835710

**Published:** 2026-07-30

**Authors:** Osama Hamad, Simon Klára, Mohammed Elmadani, Godfrey Mbaabu, Lívia Tóth, Éva Horváth, Amer Mesmar, Orsolya Máté

**Affiliations:** 1 Doctoral School of Health Sciences, Faculty of Health Sciences, University of Pécs, Pécs, Hungary; 2 Institute of Emergency Care, Pedagogy of Health and Nursing Sciences, Faculty of Health Sciences, University of Pécs, Pécs, Hungary

**Keywords:** ASD, autism, autism spectrum disorder, emergency care, healthcare, healthcare provider, nurses, quality of life

## Abstract

**Objective:**

This systematic review aims to synthesize existing evidence on emergency department (ED) experiences, service-delivery challenges, and environmental and communication factors affecting autistic people and to identify strategies and interventions reported to improve patient comfort, safety, and satisfaction.

**Methods:**

A comprehensive search was conducted across databases, including PubMed, Scopus, Web of Science, and the Cochrane Library, for peer-reviewed articles published between January 1, 2014, and December 31, 2024. Data extraction was performed using standardized forms, and quality assessment was conducted using the Mixed Methods Appraisal Tool (MMAT). Data synthesis involved narrative thematic analysis to identify key barriers and facilitators to emergency department care for autistic people. The reporting of this review follows the PRISMA guidelines.

**Results:**

Eleven studies met the inclusion criteria and revealed consistent barriers affecting autistic people in emergency departments. Across settings, communication difficulties, sensory overload, long waiting times, and limited staff preparedness were the most frequently reported challenges. These factors contributed to heightened distress, behavioral escalation, and reduced satisfaction for patients and families. Facilitators identified in the literature included ASD-specific staff training, sensory-adapted environments, structured care routines, and active caregiver involvement. While not all barriers are easily modifiable, several environmental, communication, and workflow-related factors appear potentially amenable to service-level interventions. The findings further indicate that autism-specific staff training, sensory-friendly adaptations, and caregiver-supported care approaches may improve the ED experience for autistic patients and their families.

**Discussion:**

The findings underscore the multifaceted challenges ASD patients face in EDs, exacerbated by sensory overload and communication difficulties. Addressing these issues requires a comprehensive approach, including staff education, environmental adjustments, and efficient triage systems. Engaging parents as partners in care also emerged as critical for delivering patient-centered care.

**Conclusion:**

This review emphasizes the urgent need for systemic reforms in ED care for ASD patients. Strategies such as sensory-friendly environments, enhanced staff training, and patient-centered policies can improve patient outcomes and satisfaction. Future research should focus on evaluating the long-term impacts of these interventions and identifying innovative solutions to meet the complex needs of this vulnerable population.

**Systematic Review Registration:**

https://www.crd.york.ac.uk/PROSPERO/view/CRD42024532107, PROSPERO CRD42024532107.

## Introduction

Autism Spectrum Disorder (ASD) is a complex neurodevelopmental condition characterized by persistent difficulties in social communication and interaction, alongside restricted and repetitive patterns of behavior, interests, or activities (1, 2). In recent years, autism has increasingly been understood through a neurodiversity-informed perspective, which recognizes autistic characteristics as part of natural human variation cognition rather than solely a disorder requiring correction (3, 4). As the global prevalence of autism continues to rise, ensuring equitable healthcare experiences for autistic people has become an important public health priority (5–8).

Emergency departments (EDs) are essential points of access for urgent medical, psychiatric, and behavioral care. For autistic people, however, the emergency care experience can be particularly challenging. The fast-paced, unpredictable nature of EDs, combined with sensory stimuli and communication demands, may create barriers to effective assessment, treatment, and patient engagement (9–13). These challenges are especially important given evidence suggesting that autistic people frequently require emergency services due to a range of co-occurring health needs (14–17).

Given the increasing emphasis on person-centered healthcare, there is a growing recognition that health services should be responsive to the diverse needs, preferences and capacities of the populations they serve. For people with autism, access to high-quality health care services may require adaptations to communication, sensory environments, and organizational processes that accommodate individual needs and preferences. Emerging evidence suggests that many barriers experienced by autistic individuals arise not only from personal characteristics but also from healthcare systems that are insufficiently responsive to neurodiverse communication styles, sensory profiles, and support requirements. Consequently, there is growing support for service models that emphasize accessibility, partnership, flexibility, and individualized care across healthcare settings (18, 19).

Although research has examined aspects of ED care for autistic people, the evidence remains dispersed across different age groups, settings, and study designs. A systematic synthesis is therefore needed to clarify the main barriers and facilitators affecting ED care and to identify strategies that may improve patient comfort, safety, satisfaction, and quality of care (13, 20, 21). Accordingly, this systematic review synthesizes and critically evaluates existing evidence on ED experiences among autistic people across the lifespan, with particular attention to care barriers, service-delivery challenges, environmental and communication-related factors, and reported strategies for improving emergency care.

## Background

Autism Spectrum Disorder (ASD) is a heterogeneous neurodevelopmental condition characterized by persistent differences in social communication and interaction, accompanied by restricted and repetitive patterns of behavior. Although historically recognized as a childhood condition, ASD is now understood as a lifelong developmental profile with manifestations that evolve across the lifespan. Increasing global awareness, broader diagnostic criteria, and improved detection practices have contributed to rising prevalence rates among both children and adults (5). Many adults remain undiagnosed until later life due to diagnostic overshadowing, compensatory “camouflaging” behaviors, or misattribution of autistic traits to psychiatric conditions, contributing to delayed or missed diagnoses (22).

Delayed or missed diagnoses can have significant consequences for health and well-being. Autistic adults frequently experience co-occurring mental health conditions, including anxiety, depression, and suicidality, which may mask underlying autistic traits and contribute to crisis-driven healthcare use (22, 23). In addition to psychiatric conditions, autistic people experience higher rates of epilepsy, sleep disorders, gastrointestinal problems, metabolic disorders, and other chronic health conditions compared with the general population (23).

Communication differences are a central aspect of ASD and can influence healthcare experiences across settings. Many autistic people experience difficulties with reciprocal conversation, interpreting non-literal language, or communicating symptoms in ways expected by healthcare professionals (24). When communication needs are not recognized or accommodated, misunderstandings may occur during clinical assessment and decision-making, potentially affecting the quality and safety of care. Despite the growing prevalence of autism, studies continue to report gaps in healthcare providers' knowledge and confidence in supporting autistic people, particularly within emergency care settings (25).

These layered needs intensify the challenges of acute care. Communication differences can obscure symptoms of quality and severity, delaying recognition of underlying medical problems; difficulties articulating pain may result in behavioral expressions being misattributed to psychiatric rather than somatic etiologies. In parallel, sensory sensitivities can transform routine procedures, triage, venipuncture, and vital-sign monitoring into significant stressors, elevating distress and hindering cooperation. In aggregate, ASD traits, psychiatric comorbidity, and medical complexity interact to increase diagnostic uncertainty in urgent settings.

Within this context, EDs are both critical access points and intrinsically challenging environments for autistic adults. Designed for rapid, protocol-driven care, EDs are characterized by noise, bright lighting, unpredictability, and multiple interpersonal contacts, conditions that can exacerbate sensory overload and dysregulation. Utilization patterns reflect this tension: a large U.S. cross-sectional analysis found adult ASD-associated ED visits more than doubled between 2006 and 2011, spanning psychiatric crises, non-psychiatric medical conditions, and injuries. Psychiatric presentations were notably overrepresented: 15% of ASD-related adult ED visits were for primary psychiatric complaints, compared with 4.2% among non-ASD adults (16).

Alongside clinical acuity, structural and interpersonal barriers shape ED experiences. Communication mismatches can impede accurate histories, disrupt shared understanding of symptom onset or intent, and increase the risk of premature closure. Sensory overstimulation can trigger behavioral dysregulation that may be misread as noncompliance or aggression. Compounding matters, many ED teams report limited training, tools, and confidence in recognizing adult ASD presentations, creating systemic risk for incomplete assessment or suboptimal intervention (25).

Crucially, although clear empirical evidence directly connecting preventive community measures to fewer ER visits remains scarce, timely and well-coordinated, autism-informed local care could potentially mitigate or even prevent these crisis situations. Several studies indicate that autistic people frequently encounter barriers to accessing routine healthcare, including difficulties communicating with providers, feeling misunderstood, challenges navigating appointment systems, and inadequate care coordination (26, 27). Furthermore, other studies found that individuals who engage in prolonged masking may experience cumulative exhaustion and burnout that can precipitate acute mental health deterioration, while unrecognized or inadequately managed anxiety and depression may increase the risk of suicidality, panic, or severe emotional dysregulation (16, 22, 23). Similarly, further studies reported that untreated or unrecognized mental health conditions, particularly anxiety and depression, can exacerbate psychological vulnerability and increase the risk of severe emotional dysregulation, panic episodes, and suicidality (28–30). These barriers are associated with adverse outcomes such as untreated mental health conditions, delayed treatment, and missed referrals, all of which may contribute to avoidable escalation of distress and subsequent ED use (26–28), including increased psychiatric presentations, ED visits, and hospital admissions among autistic adults (31, 32).

Accordingly, effective communication is central to safe triage and timely treatment. Many autistic adults report difficulty processing rapid or ambiguous instructions, deciphering nonverbal cues, or presenting symptoms in ways that align with ED expectations; these challenges contribute to incomplete histories and misinterpretations that derail care (24). At the same time, rapid-fire questioning, complex medical terminology, and changing communicative partners can exacerbate confusion and distress. Simple accommodations, allowing additional processing time, using direct and concrete language, offering written options, and employing augmentative and alternative communication (AAC), remain underutilized despite evidence that such strategies improve mutual understanding and reduce escalation (24).

A lifespan lens clarifies how these dynamics evolve. In childhood, ED visits are often driven by co-occurring medical issues (e.g., epilepsy, gastrointestinal symptoms), acute behavioral dysregulation, injuries, or caregiver concern about abrupt behavioral change; sensory and communication differences complicate care delivery in stimulating environments, even when pediatric EDs have relatively more autism-informed resources (compared with adult settings) (16, 25). During adolescence, increasing autonomy, shifting supports, and rising mental health needs converge with transition gaps between pediatric and adult services, elevating risk for crisis-driven ED utilization and discontinuity of care (16, 25).

By adulthood, ED utilization often reflects cumulative unmet needs, persistent comorbidities, and undiagnosed or masked conditions. Adults on the spectrum are more likely to present psychiatric crises, unexplained physical symptoms, or medical conditions concealed by communication differences. Yet adult EDs frequently lack autism-specific pathways, sensory accommodations, and staff training tailored to atypical adult presentations, shortcomings associated with longer stays and higher costs (16, 25). In this context, higher ED charges refer to higher hospital billing charges reported in administrative ED datasets, not necessarily direct out-of-pocket costs to patients (16, 33). Taken together, recurrent barriers, sensory overload, communication mismatches, limited clinician preparation, and fragmented care pathways shift in form and intensity across development, underscoring the necessity of lifespan-responsive ED strategies.

Globally, autistic people bear disproportionate health burdens beyond core diagnostic features, affecting personal development and family well-being (34). Physiological conditions, sleep disturbance, gastrointestinal problems, and epilepsy are more prevalent in autistic children than in non-autistic peers, contributing to more complex clinical needs (35). Across several countries, autistic individuals consistently report poorer health and higher rates of chronic conditions than non-autistic populations. Evidence from Australia, the United Kingdom, the United States, the Netherlands, and Canada demonstrates increased prevalence of chronic physical and mental health conditions, alongside significant barriers to healthcare access and service utilization (27, 36–39).

Mental health needs add further complexity. Anxiety and mood disorders influence behavior and treatment adherence, while sensory sensitivities, heightened responses to noise, light, and textures, and difficulties expressing pain can impede timely diagnosis and intervention in urgent settings. Consequently, a tailored, autism-informed approach is essential for equitable emergency care and quality-of-life gains (12, 40–42).

Consistent with these patterns, evidence indicates that autistic people have substantial healthcare needs throughout their lives, with studies showing a higher likelihood of utilizing emergency services compared to the general population (15, 40, 43–45). However, according to Nicholas, Muskat (13), ED care processes and environments impose unique challenges for children with ASD, encountering additional barriers to effective hospital care (46). Communication challenges, sensory sensitivities, and complex behavioral needs commonly associated with ASD can interfere with standard triage, assessment, and treatment procedures in the ED setting (47, 48). These factors highlight the need for tailored approaches to care that address the unique needs of children with ASD in emergency environments.

User experience research evokes these clinical concerns. Across Israel, Canada, the United States, the United Kingdom, and Australia, families and autistic people commonly report long waits, inadequate communication, poor care coordination, and limited staff knowledge as factors linked to lower satisfaction and avoidable distress during ED encounters (49–51). Reports from providers reinforce these findings, citing chaotic environments and communication barriers as frequent obstacles to safe, person-centered care (49, 50).

Importantly, staff expertise and targeted training may shape the quality of emergency department care for children and families presenting with autism-related needs. In this context, psychiatric expertise refers to the capacity of ED teams to recognize and manage mental health presentations and risks, including distress, self-harm, suicidality, behavioral dysregulation, and co-occurring psychiatric or neurodevelopmental conditions, with specialist support accessed when required (52–55). For autistic children and those with other neurodevelopmental conditions, psychiatric expertise includes understanding sensory sensitivities, communication differences, and how the ED environment can escalate distress (55–57). More broadly, care quality for families presenting with a child on the spectrum depends on staff understanding of autism's diverse presentations, consistent application of supportive strategies, and clear, collaborative communication among clinicians, parents, and children; this requires listening to caregivers about precipitating factors, barriers, and environmental triggers that can exacerbate symptoms in busy ED settings (46, 58, 59).

Despite increasing recognition of the challenges faced by autistic people in emergency settings, important gaps remain in the evidence base. Research has often focused on specific populations, settings, or aspects of care, limiting understanding of the broader factors that influence emergency care experiences across the lifespan. Furthermore, evidence regarding effective strategies to improve access, communication, patient satisfaction, and clinical outcomes remains fragmented. A comprehensive synthesis of the available literature is therefore needed to better understand the experiences of autistic people in EDs and to identify approaches that may support more inclusive, accessible, and high-quality emergency care.

### Rationale

Increased prevalence of ASD globally (20) has significant implications for healthcare systems, particularly in EDs, where autistic people often face unique challenges and need special care. Studies have shown that autistic people are more likely to visit EDs compared to their neurotypical peers, with some age groups having up to four times the number of ED visits. Despite EDs being designed to provide rapid and efficient care for a wide range of medical emergencies, and with this increased utilization, the chaotic and sensory-intensive environment of EDs can be particularly distressing for autistic people, who often have heightened sensory sensitivities (20).

The unpredictable nature of EDs, coupled with the high levels of sensory stimuli such as bright lights, loud noises, and frequent movement, can exacerbate the sensory sensitivities of autistic people, leading to increased anxiety, agitation, and even aggressive behaviors (21). Furthermore, communication difficulties, long waiting times, staff knowledge, and parental involvement were also found to affect ASD patients' satisfaction and quality of treatment (49, 60, 61). This gap in care can result in negative experiences for patients and their families, as well as challenges for healthcare providers in delivering effective and compassionate care.

To address these challenges, there has been a growing emphasis on developing sensory-friendly interventions in EDs. Sensory-friendly interventions aim to create a more accommodating environment for autistic people by reducing sensory overload and providing a more structured and predictable experience. These interventions can include modifications to the physical environment, such as dimming lights, reducing noise levels, and providing quiet spaces. Additionally, training ED staff to recognize and respond to the unique needs of autistic people is crucial for improving patient outcomes.

Despite the recognition of these challenges, there is a gap in the literature that comprehensively compares the satisfaction of ASD patients with emergency services provided for them and regular patients. By systematically reviewing existing studies, this study aims to bridge this knowledge gap, providing a detailed analysis of the unique barriers in EDs that affect patients with ASD. Understanding these barriers is crucial for developing targeted interventions and policies that can effectively address the specific needs of disabled populations, ultimately promoting patients' satisfaction and quality of life.

### Objectives

The primary objective of this systematic review is to synthesize existing evidence on sensory-friendly interventions in EDs for autistic people. Specifically, the review aims to:

1. Synthesize Existing Evidence on Emergency Department (ED) experiences: Describe the experiences and care processes encountered by autistic people and their caregivers in ED settings.
2. Highlight Successful Interventions: Identify common barriers and facilitators influencing ED care, including communication, sensory, environmental, organizational, and staff-related factors.
3. Provide recommendations: Synthesize reported strategies and interventions intended to improve comfort, safety, satisfaction, and quality of care for autistic people in the ED.
4. Insight into future studies: Identify gaps in the current evidence base and provide recommendations for future research, clinical practice, and service improvement.

In addition to the primary objectives, this review aims to identify gaps in current research, highlighting areas where existing studies on ED care for autistic people are lacking. This will help pinpoint specific aspects of care that require further investigation and development. The review will also explore the experiences and perspectives of both ASD patients and their caregivers in ED settings, providing valuable insights into the challenges they face and the types of support that are most beneficial. Furthermore, it will assess the impact of training programs designed for ED staff to better understand and manage the needs of ASD patients, evaluating changes in staff attitudes, knowledge, and behaviors following training.

Additionally, the review will examine the implications of current healthcare policies on providing ED care for ASD patients, identifying potential areas for policy enhancement to support ASD individuals better. Promoting interdisciplinary collaboration between various healthcare professionals, including ED staff, ASD specialists, and mental health professionals, will also be a key focus, aiming to develop comprehensive care strategies that address all aspects of ASD care in emergency settings.

By achieving these objectives, this review aims to contribute to developing targeted policies and practices that enhance the ED experience for patients with ASD and improve their treatment outcomes. This includes creating a more accommodating and supportive environment in EDs and ensuring that healthcare providers are well-equipped to meet the unique needs of ASD patients. Furthermore, the insights gained from this review will help inform future research directions, fostering a deeper understanding of effective interventions and strategies. Ultimately, these efforts will promote better health outcomes and a higher quality of care for autistic people in emergency settings.

## Methods

### Search strategy

A comprehensive and structured literature search was undertaken to identify studies examining emergency department (ED) care for individuals with autism spectrum disorder (ASD). To ensure breadth and depth, seven major electronic databases were consulted: PubMed/MEDLINE, PsycINFO, Embase, CINAHL, Scopus, Web of Science, and Cochrane Central Register of Controlled Trials. Searching multiple databases enabled the inclusion of diverse methodological approaches and minimized publication bias.

The search strategy combined controlled vocabulary (e.g., MeSH terms) and free-text keywords. Boolean operators (“AND,” “OR”), phrase searching, and truncation were applied to optimize retrieval. The following core string was adapted as needed for each database: “autism spectrum disorder” OR “autism” OR ASD AND “emergency department” OR “emergency care” OR “acute care” AND “patient experience” OR “hospital experience” OR “healthcare quality” OR “satisfaction.”

The search was restricted to studies published between January 2014 and December 2024, reflecting the period in which ED-related ASD research expanded substantially. Only articles written in English were considered. To supplement database searches, a manual screening of reference lists from key publications was conducted, ensuring the capture of additional relevant studies not indexed electronically.

### PICO framework

The review question and eligibility criteria were developed using the PICO framework to ensure a clear and systematic approach to study identification and selection (Table 1).

- Population (P): Individuals of any age with a confirmed diagnosis of autism spectrum disorder (ASD) based on recognized diagnostic criteria (DSM or ICD classifications).
- Intervention/Interest (I): Emergency department (ED) care and experiences, including clinical encounters, service delivery, communication processes, environmental factors, healthcare interactions, and quality-of-care considerations within emergency settings.
- Comparison (C): Where available, studies comparing autistic individuals with non-autistic populations or examining different ED care approaches were considered. However, the presence of a comparison group was not required for inclusion due to the exploratory nature of the review.
- Outcomes (O): Outcomes of interest included patient and caregiver experiences, communication challenges, sensory and environmental barriers, healthcare utilization patterns, staff preparedness and knowledge, quality of care, satisfaction with services, and recommendations for improving ED care for individuals with ASD.

**Table 1:** PICO framework used to guide the review.

Component	Description
Population (P)	Individuals with a confirmed diagnosis of autism spectrum disorder (ASD), regardless of age
Intervention/Interest (I)	Emergency department care, experiences, healthcare encounters, and related service delivery factors
Comparison (C)	Non-autistic populations, alternative care approaches, or no comparison group
Outcome (O)	Patient and caregiver experiences, healthcare quality, communication barriers, sensory challenges, utilization patterns, staff preparedness, and satisfaction with care

### Eligibility criteria

Eligibility criteria were established according to the PICO framework, with studies selected based on their relevance to individuals with ASD receiving care in emergency department settings and reporting outcomes related to patient experiences, healthcare delivery, or quality of care. Studies were eligible for inclusion if they examined emergency department (ED) care involving individuals with a confirmed diagnosis of autism spectrum disorder (ASD), were published between 2014 and 2024, and employed quantitative, qualitative, or mixed-methods designs. Only full-text, peer-reviewed articles were included. A language restriction to English was applied due to feasibility constraints related to translation resources and to ensure consistency in methodological appraisal across studies. However, this restriction may have resulted in the exclusion of relevant research published in other languages, and this limitation is acknowledged accordingly. To promote methodological rigor, predefined inclusion and exclusion criteria guided study selection:

### Inclusion criteria

1. Participants had a documented diagnosis of ASD based on established diagnostic systems (DSM-5 or ICD), regardless of age group.
2. Eligible studies were those that described emergency department encounters, with no restrictions on the presenting complaint.
3. Studies based on quantitative, qualitative, or mixed-methods research designs were eligible.
4. Eligible studies were published within a ten-year timeframe, specifically between 2014 and 2024. The 10-year timeframe reflects the period following major shifts in ASD diagnostic standards and modern ED care practices, ensuring that included studies align with current clinical realities.
5. Only studies in the English language were included in this study.

### Exclusion criteria

Studies were excluded if:

1. They examined populations without a confirmed ASD diagnosis.
2. They focused on non-emergency settings such as outpatient clinics, primary care, or inpatient units.
3. They were published before 2014.
4. They were review articles, commentaries, editorials, conference abstracts without full text, or dissertations.
5. The full text was unavailable after reasonable retrieval attempts.

### Screening process

The study selection followed a multi-stage screening approach aligned with PRISMA guidelines. First, all retrieved records were imported into a reference-management system, where duplicate entries were removed using both automatic and manual methods. After deduplication, two independent reviewers screened titles and abstracts to remove clearly irrelevant studies.

Full texts of potentially eligible articles were then assessed independently by the same reviewers. Disagreements at any stage were resolved through discussion, and when consensus could not be reached, a third reviewer adjudicated. Throughout the screening process, inclusion criteria were applied consistently to ensure transparency and reduce selection bias. A PRISMA flow diagram documented each step, including the number of records identified, screened, excluded, and retained for final synthesis.

### Data extraction

Data extraction was performed using a structured and piloted extraction form to ensure consistency. Extracted variables included publication characteristics such as the author, year, gender of study participants, age range of study participants, setting, number of participants, and outcomes (Table 2). In addition, information on the challenges reported and the key findings pertaining to emergency department (ED) care was extracted. Two reviewers independently extracted the data, and discrepancies were resolved collaboratively (Table 3).

**Table 2:** Author, year, gender, age range, type of study, setting, no. Of participants, outcomes being assessed, and country.

Author	Year	Gender	Age range	Type of study	Setting	No. of participants	Outcomes	Country
Garrick et al. (64)	2022	∼71% male	<18 y	Quantitative cross-sectional	EDs	421	Quality of care, behavior, sensory	Australia
Ben Natan et al. (49)	2024	Mothers, female	Exact age: *NR	Correlational quantitative study	EDs	128	Satisfaction factors	Israel
Beverly et al. (14)	2021	Male and Female	Children and adolescents 2–22 y	Retrospective, pooled, cross-sectional secondary data analysis	EDs	5.9 million	Frequent ED use	United States
Lunsky et al. (17)	2015	316 males and 80 females	Adolescents/adults 12–56 y	Quantitative study	EDs	396	Emergency service use	Canada
Vohra et al. (16)	2016	Male and Female	Adults22–64 y	Cross-sectional qualitative analysis using the Nationwide Emergency Department Sample (NEDS) from 2006 to 2011.	EDs	25527	Trends: psychiatric vs. non	United States
Bilginer et al. (65)	2023	192 boys, 32 girls	0–17 y	Qualitative study using retrospective file review and phone interviews	Tertiary ED	224	Admission causes challenges.	Turkey
Muskat et al. (60)	2015	*NR	*NR	Qualitative study using interpretive description methodology	Pediatric hospitals EDs	42	ED experiences	Canada
Nicholas et al. (61)	2016	*NR	*NR	Qualitative Study	EDs	30	Family ED experience	Canada
Zwaigenbaum et al. (50)	2016	*NR	*NR	Qualitative Study	EDs	31	Provider perspectives	Canada
Kirsch et al. (48)	2018	*NR	3–21 y	Mixed-method study	ED/urgent care	10000	Parent satisfaction determinants	United States
Niro et al. (66)	2024/25	*NR	*NR	Mixed-methods, cross-sectional, explanatory	Pediatric ED	50000	Knowledge-to-practice gaps	Canada

*NR = not reported in the abstract or readily accessible article text.

**Table 3:** Factors influencing the level of satisfaction in emergency departments (EDs) for ASD patients and their families.

Study	Challenges	Key findings
(49)	Communication Difficulties, Waiting Times, Staff Attentiveness, Coordination and Organization, Sensory Overload, Staff Knowledge	Lower satisfaction due to communication difficulties, long waiting times, lack of staff attentiveness, and sensory overload. Recommendations include raising awareness and implementing strategies to improve the ED experience.
(14)	Higher ED Utilization, Comorbidities, Access to Care, Socioeconomic Factors, Transition to Adult Care	Higher ED visits among children with ASD, especially from lower-income areas. Emphasizes the need for policy interventions and improved training for ED providers
(65)	Crowded Waiting Areas, Sensory Overload, Communication Difficulties, Behavioral Issues, Staff Awareness	Challenges include crowded waiting areas, sensory overload, and communication difficulties. Recommendations include creating quieter areas and minimizing physical restraint.
(64)	Communication Difficulties, Sensory Sensitivities, Staff Awareness, Wait Times, and Parental Anxiety.	Frequent ED visits due to communication difficulties and sensory sensitivities. Highlights the need for better staff training and sensory accommodations.
(48)	Staff Interpersonal and Communication Skills (ICSs), Waiting Times, Disruptive Behavior, Sensory Sensitivities, Parental Expectations, and Preparation.	Satisfaction influenced by staff ICSs, waiting times, and disruptive behavior. Recommendations include improving staff communication skills and managing sensory sensitivities.
(17)	High emergency service use (13%), use of restraints and sedation (23%), communication and behavioral issues, and lack of structured daytime activities.	High use of emergency services and challenges in differentiating medical and psychiatric causes. Emphasizes the need for education and support for families and clinicians.
(60)	Communication Difficulties, Sensory Sensitivities, Health-Care Team Dynamics, Waiting and Transitioning, and Flexibility of Care.	Positive experiences linked to flexible and supportive healthcare providers. Recommendations include developing toolkits and improving communication between HCPs and families.
(67)	Communication Barriers, Sensory Sensitivities, Behavioral Challenges, Lack of Training, and Resource Limitations.	The need for specialized training for emergency care providers, changes to the emergency environment, and personalized care plans for children with ASD. It also highlights involving parents and fostering collaboration among healthcare professionals.
(61)	Difficulties with the logistics of care (needing to move to different locations for various procedures), communication barriers and behavioral challenges, lack of adequate knowledge, and lack of partnership with parents. Sensory intensity and lack of distraction and long waiting times.	Effective care for children with ASD in the ED includes providing distractions like televisions and art supplies and ensuring staff are trained in ASD to interact effectively with children and families. Recommendations include involving parents in care, developing ASD-friendly resources, and managing waiting times to reduce sensory overload.
(66)	Added considerations for interacting with children and families, ED as a single touchpoint, recognizing comfort and discomfort, and the need to implement practical educational interventions.	Emphasizes the need for ongoing training and creating sensory-friendly environments.
(16)	High Rates of Psychiatric Visits (15%), Injury Visits (23.7%), Non-Psychiatric Visits (higher rates), Economic Burden (Higher hospital-reported ED charges/administrative billing charges), and Lack of Trained Professionals.	Higher ED visits and costs for adults with ASD. Highlights the need for trained professionals and comprehensive care.

### Quality assessment

Quality appraisal of the included studies was undertaken using the Mixed Methods Appraisal Tool (MMAT) (62), selected for its capacity to evaluate qualitative, quantitative, and mixed-methods research within a single, coherent framework. The MMAT allows each study to be judged against methodological standards specific to its design rather than through a universal scoring system. In keeping with MMAT guidance, the appraisal process focused on domain-level criteria rather than composite scores, enabling a more nuanced assessment of methodological strengths and limitations.

Each study was evaluated systematically using the criteria relevant to its methodological category. For qualitative studies, assessments considered whether the chosen approach aligned with the research question, whether data collection procedures were adequate, whether findings were clearly grounded in the data, whether interpretations were supported by evidence, and whether there was overall coherence between data sources, collection, analysis, and interpretation. For quantitative studies, evaluation addressed the appropriateness of the sampling strategy, the representativeness of the sample, the suitability and validity of the measurements used, whether potential nonresponse bias was accounted for, and the adequacy of the statistical analyses. For mixed-methods studies, appraisal examined whether the use of a mixed-methods design was well justified, whether qualitative and quantitative components were effectively integrated, whether interpretations reflected meaningful integration of findings, whether limitations specific to each component were acknowledged, and whether the overall integration was consistent with the study's design and aims.

To enhance clarity, domain-level judgments were recorded using the MMAT notation system: “Yes” when a criterion was clearly met, “No” when it was clearly not met, and “Cannot determine” when reporting was insufficient to allow a conclusive judgment. This approach ensured that each study's quality was described with appropriate nuance, avoiding the oversimplification associated with global classifications such as “high,” “moderate,” or “low” quality. These domain-specific evaluations were not used to exclude studies but served to contextualize the strength, credibility, and interpretability of each study's findings during the synthesis. A summary table presents the MMAT criteria met or unmet across included studies, accompanied by brief justifications to promote transparency and reproducibility (Table 4).

**Table 4:** Mixed methods appraisal tool (mmat) domain-level assessments for included studies.

Study	Study Design	MMAT Criterion 1	MMAT Criterion 2	MMAT Criterion 3	MMAT Criterion 4	MMAT Criterion 5	Justification (Summary)
Garrick et al. (2022) (64)	Quantitative cross-sectional	Yes	Cannot determine	Yes	No	Yes	Sampling strategy appropriate; representativeness unclear; valid measures used; nonresponse bias not discussed; analysis appropriate.
Ben Natan et al. (2024) (49)	Quantitative correlational	Yes	Cannot determine	Yes	No	Yes	Sampling adequate; representativeness limited; validated tools used; nonresponse bias unaddressed; statistics suitable.
Beverly et al. (2021) (14)	Quantitative secondary data	Yes	Yes	Yes	Cannot determine	Yes	The national dataset ensured representativeness, administrative measures were appropriate, nonresponse bias was indeterminate, and analyses were rigorous.
Lunsky et al. (2015) (17)	Quantitative	Yes	Cannot determine	Yes	No	Yes	Sampling aligned with aims; representativeness unclear; appropriate measures; nonresponse bias absent; suitable statistical analyses.
Vohra et al. (2016) (16)	Quantitative (NEDS analysis)	Yes	Yes	Yes	Cannot determine	Yes	National database supports representativeness; coding is appropriate; nonresponse bias is unclear; analysis is robust.
Bilginer et al. (2023) (65)	Qualitative	Yes	Yes	Yes	Yes	Yes	Qualitative design appropriate; data collection adequate; findings grounded in data; interpretations supported; coherence achieved.
Muskat et al. (2015) (60)	Qualitative	Yes	Yes	Yes	Yes	Yes	Strong alignment between method and aims; rigorous data procedures; themes supported; coherent analysis.
Nicholas et al. (2016; Cohen-Silver group) (61)	Qualitative	Yes	Yes	Yes	Yes	Yes	Appropriate approach, clear data methods, interpretations grounded, and strong methodological coherence.
Nicholas et al. (2016; Kilmer group) (61)	Qualitative	Yes	Yes	Yes	Yes	Yes	Design appropriate; data well collected; themes supported; analysis coherent.
Kirsch et al. (2018) (48)	Mixed-methods	Yes	Yes	Yes	Cannot determine	Yes	Mixed-methods justified; integration evident; interpretation coherent; component-level limitations minimally addressed.
Niro et al. (2024) (68)	Mixed-methods	Yes	Yes	Yes	Cannot determine	Yes	Mixed-methods approach justified; strong integration; interpretation adequate; limited detail on component limitations.

MMAT Criteria 1–5 correspond to the design-specific domains for qualitative, quantitative descriptive, and mixed-methods studies as outlined in the MMAT (2018). “Yes” indicates that a criterion was met, “No” indicates that it was not met, and “Cannot determine” reflects insufficient information to make a judgment.

MMAT, Mixed Methods Appraisal Tool; NEDS, Nationwide Emergency Department Sample.

### Data synthesis

The data synthesis for this systematic review on enhancing ED care for autistic people involved a comprehensive and structured approach to collate and analyze the findings from the included studies. The synthesis process provided a holistic understanding of the current state of ED care for ASD patients.

### Narrative synthesis

As the included studies varied substantially in design, measures, and outcomes, statistical pooling was not feasible. A narrative synthesis was therefore conducted following methodological guidance from the Cochrane Handbook for Systematic Reviews of Interventions. The synthesis proceeded through three steps:

1. Preliminary organization of findings into structured summary tables.
2. Exploration of patterns across studies by comparing recurring issues such as communication barriers, sensory sensitivities, waiting times, staff preparedness, and caregiver involvement.
3. Assessment of robustness, with attention to variation in study quality to avoid overinterpreting weaker evidence.

Using this approach made it possible to summarize the distribution of reported effects across diverse study types while preserving the context of each study.

### Thematic analysis

To integrate qualitative and qualitatively derived findings, a thematic analysis was carried out using the reflexive framework described by Braun and Clarke (63). A hybrid coding approach was adopted: deductive codes were informed by the review objectives, while inductive coding allowed unanticipated concepts to emerge from the data. The analysis followed six steps: familiarization with the extracted data, generating initial codes, identifying candidate themes, reviewing and refining these themes, assigning clear definitions, and integrating the final themes with the narrative synthesis. Two reviewers conducted the coding independently. Differences were resolved through discussion to strengthen consistency and reduce interpretive bias.

### Analytic process

No analytic software was used. Coding, categorization, and theme development were carried out manually using organized notes, coding tables, and hand-drawn thematic diagrams. This process ensured transparency and allowed an audit trail of analytical decisions.

### Influence of study quality

Study quality played an important role in interpreting the findings. Studies that met a higher number of MMAT criteria contributed more strongly to theme development, while findings from lower-quality studies were considered with greater caution. When conflicting results appeared, differences in methodological characteristics, such as sampling procedures, measurement clarity, and coherence of qualitative analysis, were examined to explain these discrepancies. For mixed-methods studies with limited integration, the qualitative and quantitative components were assessed separately to avoid overstating conclusions.

### Study selection

**Figure 1:**
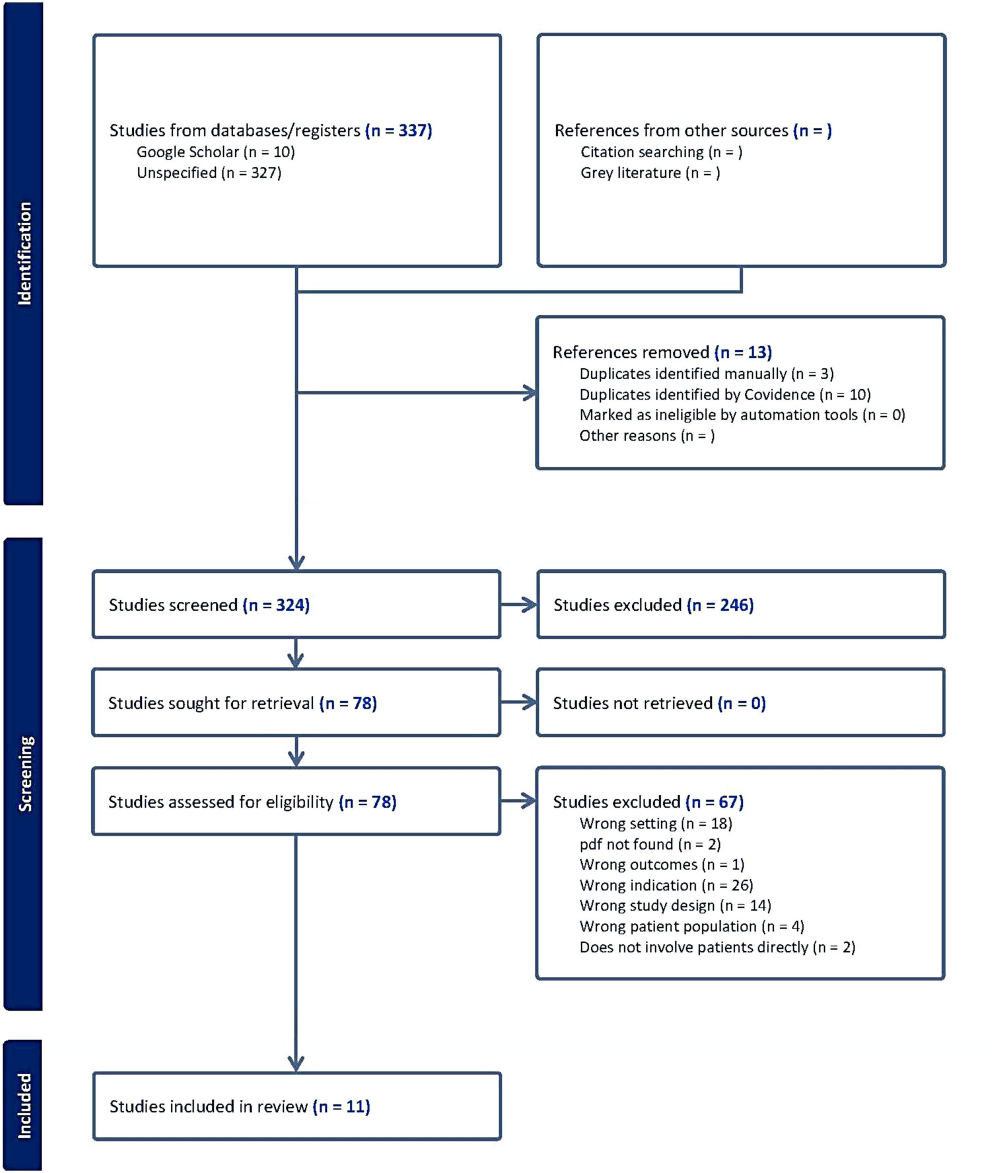
Study selection: the prisma flow diagram provides A visual summary of the study selection process.

The study selection process was documented using the PRISMA (Preferred Reporting Items for Systematic Reviews and Meta-Analyses) flow diagram (Figure 1). This documentation included records identified through database searches, additional records found from other sources, records screened, full-text articles assessed for eligibility, and the studies that were ultimately included in the final review.

## Results

A total of 337 studies were identified through database searches across PubMed/MEDLINE, PsycINFO, Embase, the Cochrane Central Register of Controlled Trials, CINAHL, Scopus, and Web of Science. After removing 13 duplicates through manual and automated processes, 324 studies were screened by title and abstract, resulting in the exclusion of 246 records that did not meet the inclusion criteria. The remaining 78 full-text articles were retrieved and assessed for eligibility, of which 67 were excluded for the following reasons: wrong setting (*n* = 18), full text not found (*n* = 2), wrong outcomes (*n* = 1), wrong indication (*n* = 26), inappropriate study design (*n* = 14), wrong patient population (*n* = 4), and studies not involving patients directly (*n* = 2). These exclusion categories correspond one-to-one with the PRISMA 2020 flow diagram. Ultimately, 11 studies met all eligibility criteria and were included in the final synthesis.

The selected studies represented diverse methodologies, including qualitative, quantitative, and mixed-method designs, and spanned multiple geographic regions, including North America, Europe, Australia, and the Middle East. These studies collectively provided a comprehensive overview of the barriers and facilitators impacting ED care for autistic people. The included studies encompassed varying age groups and highlighted distinct challenges, including communication barriers, sensory sensitivities, extended waiting times, and inadequate staff training.

Thematic analysis of the findings revealed consistent patterns across the studies. Communication challenges were identified as a critical factor influencing the quality of care. Children and adults with ASD often struggle to express their symptoms or discomfort, leading to delays in diagnosis and treatment. Furthermore, sensory sensitivities to light, sound, and odors in the ED environment were also reported to exacerbate anxiety and behavioral issues among ASD patients, highlighting the need for sensory-friendly modifications.

The findings of this review align with previous studies that have highlighted the unique challenges faced by autistic people and their families in EDs. For instance, earlier research has shown that children with ASD experience communication difficulties with healthcare providers in EDs, which significantly predicts lower satisfaction (49, 64, 65). Additionally, staff awareness has been identified as a crucial factor affecting patient satisfaction (49, 64). Moreover, studies have indicated that the needs of mothers and children with ASD, as well as responses to mothers' questions and concerns, are often inadequately addressed in the ED (49).

The presence of an ASD-sensitive environment in the ED has also been identified as a significant factor influencing satisfaction, particularly for patients with sensory sensitivities and behavioral needs (14, 64, 65). Furthermore, long waiting times for examinations by both nurses and physicians have been associated with the level of satisfaction (49, 65). This review contributes to the existing body of literature by identifying and addressing the primary challenges faced by ASD patients and their families in EDs, which significantly impact their level of satisfaction.

Challenges and Their Impact on Satisfaction in Emergency Departments for ASD Patients and Their Families

### Communication difficulties

Communication difficulties are a significant challenge for children with ASD in ED settings, impacting the quality of care and satisfaction levels of both patients and their families. According to Ben Natan, Igbarin (49), communication difficulties were a significant predictor of lower satisfaction with ED care for families of children with ASD. These difficulties often manifest as an inability to effectively express needs, pain, or discomfort, leading to misunderstandings and inadequate care (49, 64, 65). The high-stress environment of the ED exacerbates these communication barriers, as children with ASD may struggle to interpret questions and instructions, further complicating their interactions with healthcare providers (60). Additionally, the lack of staff training and awareness regarding ASD-specific communication needs often results in frustration and anxiety for both the children and their parents (64, 67). According to Kirsch, Meryash (48), and Lunsky, Paquette-Smith (17), addressing these communication challenges through targeted training for ED staff and the implementation of ASD-friendly communication strategies is crucial for improving the overall ED experience for children with ASD and their families.

#### Sensory overload (environment)

Sensory overload in ED environments is a significant challenge for children with ASD, contributing to increased anxiety and disruptive behaviors. The intense sensory stimuli commonly found in EDs, such as bright lights, loud noises, and strong odors, can be overwhelming for these children, exacerbating their sensory sensitivities (48, 49, 60, 64). This heightened sensory environment often leads to significant distress, making it difficult for children with ASD to cope and cooperate during medical procedures (60, 65). The lack of appropriate sensory accommodation and the general unawareness of healthcare providers regarding these sensory challenges further aggravate the situation, resulting in lower satisfaction with care among families (64, 67). According to Kirsch, Meryash (48), and Ben Natan, Igbarin (49), addressing sensory overload through environmental modifications, such as creating quieter and dimmer areas, and enhancing staff training on sensory sensitivities are crucial for improving the ED experience for children with ASD and their families.

#### Long waiting times

Extended waiting periods in the ED are particularly challenging for children with ASD and their families and significantly impact on the satisfaction levels of families with children who have ASD. Long waiting periods exacerbate anxiety and behavioral issues in children with ASD, who often struggle with the unpredictability and sensory overload of the ED environment (48, 49, 64, 65). These prolonged waits can lead to increased distress and disruptive behaviors, making it challenging for healthcare providers to deliver effective care (Bilginer et al., 2023; Kirsch et al., 2018). Parents' perceptions of care are also negatively affected by long waiting times, as they often feel that their child's needs are not being prioritized (61, 64). Ben Natan, Igbarin (49) and Muskat, Burnham Riosa (60), reported that addressing this issue requires implementing strategies to reduce waiting times and improve the overall efficiency of ED processes, thereby enhancing the care experience for children with ASD and their families (Ben Natan et al., 2024; Muskat et al., 2015).

#### Staff awareness, knowledge, and training

Staff awareness, knowledge, and training are critical factors influencing the quality of care and satisfaction levels in EDs for children with ASD. Insufficient staff knowledge about ASD-specific needs often leads to inadequate care and increased anxiety for both children and their families (49, 64, 65). Many healthcare providers lack the necessary training to effectively communicate with and manage the behaviors of children with ASD, which can result in misunderstandings and suboptimal care (14, 48). Studies emphasize the importance of specialized training programs to enhance staff competence in handling ASD-related challenges, including communication difficulties and sensory sensitivities (66, 67). According to Garrick, Lee (64), and Muskat, Burnham Riosa (60), implementing comprehensive training and awareness initiatives can significantly improve the ED experience for children with ASD and their families, fostering a more supportive and understanding healthcare environment.

#### Coordination and organization

A well-coordinated and organized ED can make a significant difference in the experience of families with children with ASD. Ben Natan, Igbarin (49) highlight that a lack of coordination and cooperation among ED staff and disorganized work processes significantly impacts the quality of care provided to these patients, which can lead to delays and confusion, exacerbating the stress and anxiety experienced by children with ASD and their parents, leading to more challenging behaviors and lower overall satisfaction with the care received. Effective coordination, clear communication among healthcare providers, and streamlined organizational practices are essential to ensure that the unique needs of children with ASD are met promptly and efficiently, thereby improving their ED experience and outcomes. Implementing structured protocols and fostering teamwork among healthcare providers can mitigate these issues, enhancing the overall care environment for ASD patients and their families (49).

#### Behavioral challenges

Behavioral responses associated with ASD, such as heightened distress or difficulty coping with unfamiliar environments, can intensify the challenges experienced during ED encounters for both the child and healthcare providers. These challenges include heightened anxiety, aggression, self-injury, and disruptive behaviors, which can be triggered by the unfamiliar and often overstimulating environment of the ED (48, 64, 65). Sensory sensitivities to lights, sounds, and smells further contribute to these behavioral issues, making it difficult for children with ASD to cope with the stress of medical procedures (60). Additionally, the lack of structured daytime activities and previous negative experiences in the ED can lead to recurrent admissions and increased behavioral problems (17). Effective management of these behavioral challenges requires healthcare providers to have a deep understanding of ASD and to implement individualized care plans that address each child's specific needs and triggers (67). Enhanced staff training and a more sensory-friendly environment are crucial in improving the ED experience for children with ASD and their families (48, 64).

#### Parental involvement

Involving parents in the care process is essential for improving satisfaction levels in the ED. Parents often serve as advocates and translators for their children, providing essential information about their child's baseline behavior, needs, and effective coping strategies (60). Their involvement is vital for ensuring that healthcare providers understand and accommodate the unique needs of each child with ASD. Studies have shown that when parents are actively involved in the care process, it leads to better communication, reduced anxiety for the child, and overall improved satisfaction with the care received (67). However, parents frequently report feeling excluded from the care process and that healthcare providers often dismiss their insights (61). To enhance the ED experience for children with ASD and their families, it is essential to recognize and respect parents as partners in care, valuing their expertise and involving them in decision-making processes (60, 67).

## Discussion

### Summary of principal findings

This review identified recurrent barriers shaping emergency care experiences for autistic people across the lifespan, including communication mismatches, sensory stressors in high-stimulus environments, prolonged waiting and throughput delays, and limited healthcare professionals' preparation specific to autism. Importantly, many of these barriers reflect modifiable characteristics of healthcare systems rather than characteristics inherent to autistic individuals themselves. From a person-centered care perspective, the findings suggest that emergency departments often fail to adequately adapt communication processes, care environments, and service-delivery models to the needs of neurodivergent patients. These shortcomings can reduce accessibility, responsiveness, and equity within emergency care services. Conversely, the evidence indicates that autism-informed communication approaches, sensory-adapted environments, staff training, and caregiver and family partnership represent practical system-level strategies for improving patient-centered care and enhancing the quality of emergency services for autistic people and their families.

### Interpretation in the context of the literature

Study synthesis supports a systems-interaction perspective: many of the behaviors observed in emergency settings are context-responsive expressions to environmental and communicative stressors, rather than intrinsic “problems” within the child or adult. Studies indicate that sensory load (noise, lighting, crowding, unpredictability) and communication pace/ambiguity as proximal triggers of distress and escalation; these triggers are well-documented in quality-improvement and scoping reviews that propose structured toolkits, low-stimulus spaces, and workflow adjustments to reduce overstimulation (11).

From a person-centered care perspective, these findings suggest that many challenges experienced by autistic people arise not only from individual characteristics but also from a mismatch between patient needs and healthcare system design (18). This view aligns with emerging literature that conceptualizes healthcare barriers as consequences of environmental, organizational, and communication-related obstacles that limit the accessibility of services for autistic individuals. This interpretation supports a shift from deficit-focused approaches toward more responsive models of care that adapt communication practices, environmental features, and service delivery processes to individual needs. Such adaptations may enhance accessibility, inclusion, and equity while promoting more positive care experiences for autistic people across the lifespan (18, 19).

Beyond individual encounters, organizational design matters. Hospitals pursuing “autism-friendly” initiatives report the need to operationalize the term through concrete changes spanning people, place, and time, including flexible scheduling/flow, environmental control options, and routine caregiver involvement, rather than relying on *ad hoc* accommodations (68).

### What works in clinician training and why

Evidence from mixed-methods and implementation studies suggests that training improves ED care for autistic people when programs include four recurring components:

1. Autism-specific communication strategies [clear, concrete language; slower pacing; visual supports; and alternative and augmentative communication (AAC)]
2. Sensory support and de-escalation techniques (identifying triggers; offering regulation tools; sequencing procedures predictably);
3. Recognition of atypical distress and pain expression (to avoid diagnostic overshadowing and unnecessary restraint/sedation); and
4. Structured family partnership (explicitly inviting caregiver expertise into planning and consent for adjustments).

Programs that pair didactics with simulation/case-based coaching (e.g., Project ECHO Autism hubs; emergency-provider curricula integrating pediatric emergency medicine and developmental pediatrics) report improvements in provider confidence and self-efficacy, better caregiver-rated experiences, and fewer escalations requiring restrictive measures. Sensory toolkits and environmental checklists embedded within training appear to be the highest-yield “bridging” tools because they translate knowledge into immediate, visible changes at the bedside (66). Notably, ED teams consistently request ongoing refreshers and workflow-integrated prompts rather than one-off lectures, underscoring the importance of longitudinal reinforcement and interprofessional formats (nurses, physicians, child life, social work, and security) for sustained impact (66).

Beyond improving clinical knowledge and skills, autism-specific training may facilitate the delivery of person-centered care by equipping healthcare professionals to recognize and respond to individual communication preferences, sensory needs, and behavioral expressions. In doing so, training helps shift practice away from deficit-oriented interpretations of autistic behaviors toward a more contextual understanding of patient needs. This approach may enable emergency departments to provide more responsive, accessible, and inclusive care while reducing the risk of misunderstanding, distress, and inequitable healthcare experiences.

### System-level strategies to reduce ED waits (and why they matter for autism)

Prolonged and unpredictable waiting times can intensify sensory overload and psychological distress in autistic people. Therefore, measures aimed at improving patient flow should not be viewed solely as operational improvements but also as essential clinical safety strategies for this population. Multiple systematic reviews and multi-site studies show that the following process redesigns reliably reduce door-to-provider time, length of stay (LOS), and “left without being seen” rates:

- Fast-track/streaming models for lower-acuity presentations;
- Provider-in-triage (PIT)/team triage that initiates diagnostics and disposition earlier;
- Split-flow intake with rapid assessment zones.

Across heterogeneous settings, these interventions are associated with modest-to-substantial LOS reductions and improved flow; PIT and fast track are repeatedly singled out as effective and generally cost-neutral. Pediatric ED and urgent-care implementations demonstrate meaningful decreases in door-to-provider time and LOS after fast-track adoption (69).

Complementary strategies, Lean/Six Sigma for waste reduction and standardized pathways, real-time operational dashboards integrated with the EHR, and electronic triage decision support can further compress bottlenecks and create predictability. Although evidence quality varies, studies report shorter waits, fewer delays, and better situation awareness for clinical teams following these approaches (70).

Importantly, these interventions should be viewed not only as operational improvements but also as person-centered service redesign strategies. Reducing waiting times and improving care predictability may enhance accessibility for autistic patients, whose experiences are often disproportionately affected by uncertainty, sensory overload, and prolonged exposure to crowded emergency environments.

#### Implications for autistic people

Given the sensitivity to time in crowded spaces and unexpected transitions, EDs should consider neurodiversity-informed “accelerated triage” flags within existing split-flow or PIT models (e.g., early rooming to low-stimulus spaces, cueing the team to use visual supports). While direct trials of autism-specific fast-track are limited, converging evidence from sensory-friendly QI projects and scoping reviews supports prioritizing wait-time reduction as a core accommodation (11).

### Environmental design and low-stimulus care pathways

Multiple QI and design-thinking studies emphasize low-stimulus care pathways (quiet rooms, dimmable lighting, noise attenuation, clear wayfinding, and procedure sequencing) as first-line, non-pharmacologic measures that reduce escalation risk and improve caregiver-rated experiences. Embedding these features into standard ED layout and kits, rather than relying on availability of *ad hoc* spaces, improves uptake and equity (11).

Such environmental adaptations reflect a core principle of person-centered healthcare by acknowledging and accommodating individual sensory needs. Rather than requiring autistic patients to tolerate environments that may be distressing or inaccessible, these approaches seek to create healthcare settings that are flexible, inclusive, and responsive to neurodiversity.

### Family partnership as a routine practice, not a contingency

Across pediatric and mixed-age evidence bases, inviting caregiver expertise (baseline behaviors, triggers, and effective supports) and shared decision-making correlate with smoother encounters and fewer misunderstandings. Incorporating caregiver expertise into routine emergency care reflects key principles of person-centered care, including shared decision-making, respect for individual preferences, and collaborative care planning. The findings therefore support caregiver partnership as an essential feature of responsive health-service delivery rather than an optional component of care.

### Gaps and priorities for future research

Three gaps remain prominent. First, autism-specific pathways in adult emergency departments remain less developed than those available in pediatric settings, despite the growing recognition of autism in adulthood and the complex health profiles often seen in this population (67, 71). In pediatric emergency care, autism-informed approaches typically involve identifying autism or related neurodevelopmental needs at triage, gathering caregiver input about communication preferences and sensory triggers, providing access to quieter spaces or sensory-support resources, using visual aids, sequencing procedures predictably, and involving child-life or developmental specialists where available. These measures aim to reduce distress by adapting the environment, communication style, and clinical processes to the child's individual needs. Comparable pathways for autistic adults, however, are less consistently described in the literature, and adult ED services may lack structured procedures for recognizing autism, modifying communication, addressing sensory sensitivities, or incorporating input from caregivers or support persons (64). This gap is increasingly important given rising autism diagnoses among adults, particularly those aged 26–34 years, and the high prevalence of co-occurring mental, physical, and sensory health conditions that may complicate emergency assessment and management (72, 73).

Second, most studies evaluate acceptability and short-term process outcomes; prospective, controlled evaluations of training and system redesigns (e.g., fast track with autism flags, PIT+sensory kits) are needed to quantify effects on safety events, escalation, sedation/restraint use, and return visits (56, 64, 74–76). This is supported by a recent study in which both healthcare providers and parents emphasized the importance of sensory-adapted spaces and identified inadequately designed hospital environments as a major barrier to providing optimal care for autistic patients (76).

Third, implementation of science questions, including how to sustain staff training, prompt consistent use of autism-specific toolkits, and scale environmental adjustments, requires further attention, particularly in resource-constrained EDs where space, staffing, and sensory modification options may be limited (31, 64, 72). Research has identified persistent training gaps among ED staff and a need for ongoing education, yet little is known about how best to sustain training effects over time (56). Likewise, while sensory toolkits and environmental modifications have demonstrated feasibility and acceptability, there is insufficient evidence on effective methods for promoting their sustained use, embedding them within organizational workflows, and scaling these interventions across diverse and resource-constrained EDs where staffing, space, and infrastructure limitations may impede implementation (9, 11, 77).

### Limitations of current emergency department models

Current emergency department (ED) models present several systemic limitations that disproportionately affect autistic people. Most EDs remain highly sensory-intense environments, characterized by bright lighting, noise, and unpredictable flow, despite evidence that sensory-adaptive spaces and toolkits improve patient comfort and cooperation. Clinicians often receive limited autism-specific training, leaving gaps in communication strategies, sensory-support skills, and recognition of atypical distress responses, as shown in studies where fewer than half of ED staff reported mandatory ASD training. Standard triage and throughput systems focus on medical acuity rather than sensory vulnerability, and although fast-track and provider-in-triage models can reduce wait times, these approaches are rarely adapted for neurodivergent needs. Additionally, ED workflows frequently underutilize caregiver expertise, and real-time operational tools that could mitigate sensory- and waiting-related distress are not widely implemented. Collectively, these limitations highlight the need for autism-informed design, workforce training, and system-level reform within emergency care (68).

### Strength of the evidence and risk-of-bias considerations

Overall, the strength of evidence supporting the key findings of this review is best characterized as low to moderate, reflecting the predominance of observational and qualitative designs and the methodological variability across studies. Although the MMAT appraisal indicated that several studies met most design-specific criteria, others demonstrated methodological limitations, particularly in sampling rigor, measurement clarity, and integration of qualitative and quantitative components. Importantly, a sensitivity-style reading of the synthesis suggests that the major themes (communication barriers, sensory overload, staff preparedness, and wait-time challenges) were not driven by the lowest-quality studies; rather, these themes appeared consistently across studies with higher MMAT ratings. However, the small number of studies included and their concentration in high-income settings reduce confidence in the generalizability of conclusions. Taken together, the available evidence suggests that interventions such as staff training, sensory-responsive environments, and structured communication strategies are consistently reported as contributing to improved comfort and satisfaction, although the evidence remains observational.

### Strengths and limitations

This systematic review has several strengths, including its comprehensive search strategy across multiple databases, clearly defined inclusion criteria, and the use of standardized data extraction and quality assessment tools. These methodological features enhance the rigor and transparency of the synthesis. However, there are important limitations that warrant consideration. One of the main limitations of this review is the small number of eligible studies (*n* = 11), the majority of which were conducted in high-income countries. This concentration of evidence restricts the cultural and structural diversity of emergency care contexts represented in the literature and limits the generalizability of the findings to low- and middle-income settings; accordingly, the conclusions should be interpreted with caution when considering the global applicability of autism-informed ED practices. In addition, the heterogeneity of study designs and outcomes may reduce comparability across studies and contribute to interpretive complexity. Potential publication bias also cannot be ruled out, particularly given the reliance on published, English-language studies, which may have excluded relevant research from non-English-speaking regions. Finally, limiting the review to accessible full-text publications may have led to the omission of valuable insights from unpublished or context-specific reports.

### Implication for practice

The findings of this review have implications not only for healthcare professional's individual but also for the design and organization of emergency care systems. Consistent with the principles of person-centered health and care systems, improving emergency experiences for autistic people requires services to adapt to individual needs through flexible communication approaches, sensory-responsive environments, collaborative decision-making, and organizational practices that promote accessibility and equity.

Improving emergency department (ED) care for people with autism requires a shift from *ad hoc* accommodations toward structured, system-level adoption of autism-informed practices. The evidence indicates that meaningful improvements arise when EDs combine clinician training, environmental adaptation, and operational redesign.

First, autism-specific clinician training should be embedded as a standard competency for physicians, nurses, and ancillary staff. Effective programs consistently include targeted instruction in communication strategies, sensory-support techniques, and recognition of atypical pain or distress expression, along with simulation-based or case-based coaching to reinforce skills. Such approaches have been associated with improved provider confidence and more positive caregiver experiences in ED settings. Training that integrates practical tools, such as sensory kits, communication boards, and structured de-escalation protocols, can further support consistent implementation (78).

Second, the literature clearly shows that environmental and sensory modifications enhance the accessibility of EDs for autistic children and adults. Evidence from quality-improvement projects and design-thinking studies underscores the value of low-stimulus spaces, adjustable lighting, noise reduction, and predictable procedure sequencing, which reduce escalation risk and facilitate cooperation during care. Sensory-adaptive rooms and standardized sensory toolkits have demonstrated high acceptability among both families and healthcare professionals. Embedding these modifications into routine ED workflows ensures equitable access rather than relying on the availability of specialized staff or isolated rooms (68).

Third, the review highlights that addressing throughput and wait time barriers is crucial for mitigating sensory overload and distress. System-level strategies such as fast-track pathways, provider-in-triage models, and split-flow intake processes have repeatedly been shown to reduce door-to-provider time, length of stay, and crowding, interventions that indirectly but substantially benefit autistic people who are disproportionately affected by long, unpredictable waits. These operational improvements can be complemented by real-time dashboards and Lean approaches that enhance situational awareness, streamline decision-making, and help staff anticipate bottlenecks that may exacerbate patient distress (79).

Lastly, strengthening family partnership should be viewed as a core clinical practice rather than an optional accommodation. Caregivers hold essential knowledge about triggers, communication preferences, and regulation strategies; involving them early in triage and throughout care planning improves alignment, reduces misunderstanding, and supports safer encounters.

Collectively, these implications underscore the need for EDs to adopt integrated, multidisciplinary approaches that combine trained staff, sensory-responsive environments, caregiver collaboration, and operational efficiency. Implementing these changes can create more predictable, humane, and equitable emergency care experiences for autistic people across the lifespan.

## Conclusion

This review demonstrates that improving emergency department care for autistic people requires coordinated attention to communication, sensory experience, and operational design. The findings suggest that many challenges encountered by autistic people in emergency departments are related to healthcare-system factors that may be modified, highlighting the importance of person-centered service delivery and system-level adaptation. From this perspective, improving care involves redesigning communication practices, care environments, and clinical workflows to better accommodate neurodivergent needs, rather than focusing solely on individual patient characteristics. However, these barriers vary in their degree of modifiability, and some require broader organizational and system-level change. Accordingly, interventions that incorporate autism-informed communication strategies, sensory-responsive practices, and streamlined care pathways show promises for improving comfort, safety, accessibility, and equity within emergency care settings (68).

Beyond highlighting persistent barriers, the findings point to opportunities for system-level improvement. Implementation studies suggest that structured staff preparation, sensory-adaptive environments, and process improvements, such as fast-track models and real-time operational supports, may help reduce distress triggers and align ED routines more closely with the needs of neurodivergent patients. Further rigorous, multisite research is needed to determine which approaches are most effective, sustainable, and transferable across diverse emergency care settings (79).

More broadly, the findings reinforce the value of person-centered health system redesign. Many of the barriers identified in this review were linked to modifiable aspects of healthcare delivery, including communication practices, environmental conditions, organizational processes, and staff preparedness. Addressing these factors through autism-informed and neurodiversity-affirming approaches may enable emergency departments to become more responsive, inclusive, and equitable, while promoting meaningful partnerships with autistic people and their families.

Ultimately, this review contributes to a growing recognition that autism-informed emergency care is both feasible and essential. Aligning ED systems with neurodiversity-affirming principles offers a pathway toward more humane, predictable, and effective care for autistic people across the lifespan.

## Data Availability

The original contributions presented in the study are included in the article/Supplementary Material, further inquiries can be directed to the corresponding author.
